# Reduction in eye emergency attendance and the correlation between the COVID-19 and conjunctivitis diagnoses during the COVID-19 pandemic

**DOI:** 10.5935/0004-2749.202100115

**Published:** 2021

**Authors:** David Leonardo Cruvinel Isaac, Leopoldo Magacho, Eduardo Akio Pereira I, Karime Ortiz Fugihara Iwamoto, Aline Rabelo Ferreira, Walton Martins Ferreira Costa Falone, Marcos Pereira de Ávila

**Affiliations:** 1 Ophthalmology Department, Centro de Referência em Oftalmologia, Universidade Federal de Goiás, Goiania, GO, Brazil

Dear Editor,

During the coronavirus disease (COVID-19) pandemic, studies have described an overall reduction in the numbers of patients visiting ophthalmology emergency departments and cases of infectious conjunctivitis (IC) in different countries^(1,2)^.

Similar to COVID-19, IC is also a highly transmissible group of diseases caused mainly by viruses^(3)^. Its transmission mode shares many similarities with the COVID-19 transmission routes^(4)^. Therefore, all suggested distancing measures could prevent or reduce the incidence rates of both diseases, but not those of some other ocular emergencies.

A retrospective cross-sectional study was conducted to compare the incidence of ocular emergencies between the COVID-19 pandemic and the same periods in the 2 years before the pandemic and to study the relationship between the numbers of IC and COVID-19 cases in a tertiary university hospital in Goiás, Brazil. Data were collected from all medical charts of patients examined between June 15 and September 15, 2018, 2019, and 2020. Institutional review board approval was obtained. The study adhered to the tenets of the Declaration of Helsinki.

**Figure 1:**
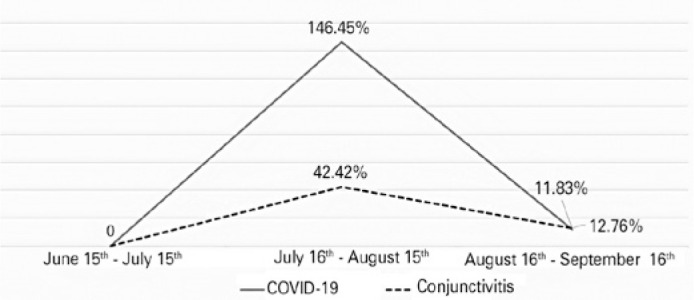
Percent variations in new diagnoses of COVID-19 and conjunctivitis compared with those in previous months.

In 2018, 2019, and 2020; 2607, 2824, and 1717 first visits to the ophthalmology emergency department were recorded, respectively. The diagnoses and frequencies are shown in table 1. The correlation between the prevalence of COVID-19 and that of IC is illustrated in figure 1.

**Table 1:** Ocular emergency diagnoses in 2018, 2019, and 2020 during the 3-month evaluation period

Diagnosis	2018 (n=2607)	2019 (n=2824)	2020 (n=1717)
Infectious conjunctivitis	353 (13.5%)	579 (20.5%)	133 (7.7%)
Allergic conjunctivitis	77 (3.0%)	73 (2.6%)	42 (2.4%)
Blepharitis/hordeolum/ dry eye	809 (31.0%)	737 (26.1%)	476 (27.8%)
Corneal foreign body	366 (14.0%)	400 (14.2%)	386 (22.5%)
Ocular trauma	115 (4.4%)	97 (3.4%)	95 (5.5%)
Uveitis	29 (1.1%)	48 (1.7%)	59 (3.4%)
Scleritis/episcleritis	15 (0.6%)	32 (1.1%)	22 (1.3%)
Retinal detachment	29 (1.1%)	32 (1.1%)	12 (0.7%)
Vitreous hemorrhage	20 (0.8%)	16 (0.6%)	17 (1.0%)
Posterior vitreous detachment	6 (0.2%)	23 (0.8%)	10 (0.6%)
Other diagnoses	788 (30.2%)	787 (27.9%)	465 (27.1%)
Total	2607 (100%)	2824 (100%)	1717 (100%)

The study shows decreases of 34.1% and 39.2% in the total number of eye emergencies in our center during the COVID-19 pandemic and the period from 2018-2019, respectively (p<0.001). Similar studies have shown reductions in the overall number of patients with eye emergencies, such as 32% in Boston, USA^(5)^, and 53% in Leicester, UK^(1)^.

A statistically significant reduction in the incidence of IC was observed in 2020 as compared with both 2018 and 2019 (p<0.001), as was described previously^(1,2,5)^, which suggests that the measures to stop the transmission of severe acute respiratory syndrome coronavirus 2 (SARS-CoV-2) infection played an indirect role in reducing the spread of IC. The incidence of other diagnoses such as uveitis (p<0.001) and corneal foreign body (CFB; p<0.001) presented significant increases in 2020 as compared with the previous years. Furthermore, no significant differences in the incidence rates of allergic conjunctivitis, retinal detachment, vitreous hemorrhage, and posterior vitreous detachment were found. A statistically significant increase in the relative percentages of ocular trauma and CFB and similar absolute numbers among the studied years may suggest that labor activities continued normally, without lockdowns, as the conditions are widely related to occupational trauma.

Considering the 133 IC diagnoses made during the 3-month evaluation period in 2020, two patients had simultaneous symptoms of COVID-19 and a positive polymerase chain reaction (PCR) test result for SARS-CoV-2. Among the patients hospitalized for COVID-19 in Hubei Province, China^(6)^, 31.6% also had conjunctivitis. More than 2 of the 133 patients with IC might have had an undiagnosed COVID-19, with conjunctivitis being the sole manifestation or initial presentation of the disease, as the patients did not present with systemic symptoms at the time of the visit. Routine reverse transcription PCR (RT-PCR) was not requested, so some patients might have also had an undiagnosed COVID-19.

Considering the similarity in transmission and incubation periods, we evaluated the possible correlation between COVID-19 and presumed IC diagnosis. Despite the borderline significance (p=0.09), a very strong, almost perfect, correlation (*r*=0.99) was found between the numbers of diagnoses of the two diseases. In other words, 98% of this correlation is only explained by the interaction between the two variables; that is, a COVID-19 diagnosis has a strong tendency to increase when the incidence of IC increases and vice versa. This result was observed to be due to the similar increasing incidence rates of both diseases during the 3-month evaluation period, which is shown in the percentual variation of cases during the period (Figure 1).

The significantly lower number of IC cases during the 2020 pandemic might have occurred owing to the social distancing and enhanced hygiene measures, which might have reduced the IC microbial agents and SARS-CoV-2 virus transmission. A trend of similar curves was observed for both infections during the pandemic, which suggests that the increase or decrease in the curve may be associated with the tightening or relaxing of the social distancing and hygiene measures such as washing hands and avoiding touching of the face with non-sanitized hands.
